# Clinical outcomes of doubled-suture Nice knot augmented plate fixation in the treatment of comminuted midshaft clavicle fracture

**DOI:** 10.1186/s12893-021-01274-4

**Published:** 2021-05-28

**Authors:** Gui Wu, Yao-qing Chen, Chun-yong Chen, Zhang-xiong Lin, Qi-yang Xie, Jun-jian Ye, Yun Xie

**Affiliations:** Department of orthopedics, First affiliated hospital, Fujian Medical University, Fujian 350005 Fuzhou, China

**Keywords:** Doubled-suture Nice knot, clavicle fractures, free bone fragments, implant failure, Plate screws fixation

## Abstract

**Background:**

Free bone fragments were difficult to be fixed in many comminuted midshaft clavicle fractures, and the absence of cortical alignment in comminuted fractures had direct influence on the stability of fixation. This survey was performed to assess the efficacy of doubled-suture Nice knot augmented plate fixation in the treatment of comminuted midshaft clavicle fractures.

**Methods:**

Between 2013 and 2018, all patients with comminuted midshaft clavicle fractures treated with doubled-suture Nice knot augmented plate fixation were retrospectively reviewed and included in this research. Demographic data of the patients, characteristics of the fractures, intraoperative parameters and follow-up data of the patients were evaluated and summarized.

**Results:**

A total of 56 patients were included in this study. The mean follow-up time was 25.6 months (range, 12–60 months). The number of male patients was 38 (67.9 %) and of the female patients was 18 (32.1 %). The average age of all patients was 47.89 ± 16.5 years. The mean time of surgery was 85.6 ± 24.0 min. The average length of incision was 9.2 ± 1.9 cm. The number of doubled-suture Nice knot applied ranged from 1 to 5 knots. All the patients reached bone union after the treatment. There was no implant failure or neurovascular injury observed. And most of the patients showed good functional outcome.

**Conclusions:**

The doubled-suture Nice knot could provide reliable fixation for small bone fragments in comminuted clavicle fractures. Combination of the doubled-suture Nice knot and plate screws fixation was a safe and effective method in comminuted midshaft clavicle fractures treatment.

## Background

About 80 % of clavicle fractures occur in the midshaft region [1]. The optimal treatment strategy of midshaft clavicle fractures is still controversial. Most of midshaft clavicle fractures can be treated conservatively. However, the frequency of operative treatment for clavicle fractures has been increased over the past years [2, 3]. The aim of the surgery is to reach early mobilization and prevention of malunion or nonunion. However, to reach those achievements, a proper fracture fixation is important.

Plate-screws fixation and intramedullary(IM)nail fixation are two most frequently used fixation methods for clavicle fractures [4, 5]. Plate-screws fixation seems to form a stronger structure than IM fixation in terms of stiffness [6, 7]. One of the difficult situations is the fixation of additional free bone fragments when the comminuted clavicle fracture is treated using plate and screws. Some surgeons simply use bridging plate techniques to connect the fractures and leave the additional free bone fragments undisturbed [8, 9]. However, the fixation stability for both IM fixation and plate fixation can be affected by the absence of cortical alignment in comminuted fractures directly [2]. Thus, some others also use screws to fix the free bone fragments after reduction. Nevertheless, in comminuted fractures, the bone fragments are usually too small to allow screw fixation [6].

A doubled-suture Nice knot, described by Boileau et al. and named after the city Nice in France, where this technique was first described [10], has unique advantages in bundling hard objects such as bone fragments. Moreover, the doubled-suture Nice knot is not easy to slip, compared with traditional flat non-sliding knots [11]. The doubled-suture Nice knot combining a doubled suture with a sliding knot is self-stabilized, solid and easy to be performed. Although it is a convenient technique, its reliability in fixing bone fragments remains largely unknown. Moreover, whether application of doubled-suture Nice knot cerclage can affect the bone union needs to be validated. In this research, the authors assess the efficacy of doubled-suture Nice knot in the auxiliary fixation of plate-screws fixation for displaced comminuted midshaft clavicle fractures.

## Patients and methods

We retrospectively reviewed patients with clavicle fractures treated in the First Affiliated Hospital of Fujian Medical University from 2013 to 2018. The inclusion criteria were: [1] unilateral acute comminuted midshaft clavicle fractures [2]; the shoulder function should be normal before the injury and no other combined injuries [3]; the internal fixation method should be locking plate-screws plus doubled-suture Nice knot [4]; at least 1-year follow-up. The general information of the patients was summarized. The classification of clavicle fractures was based on the AO/OTA fracture and dislocation classification compendium-2018.

**Fig. 1:**
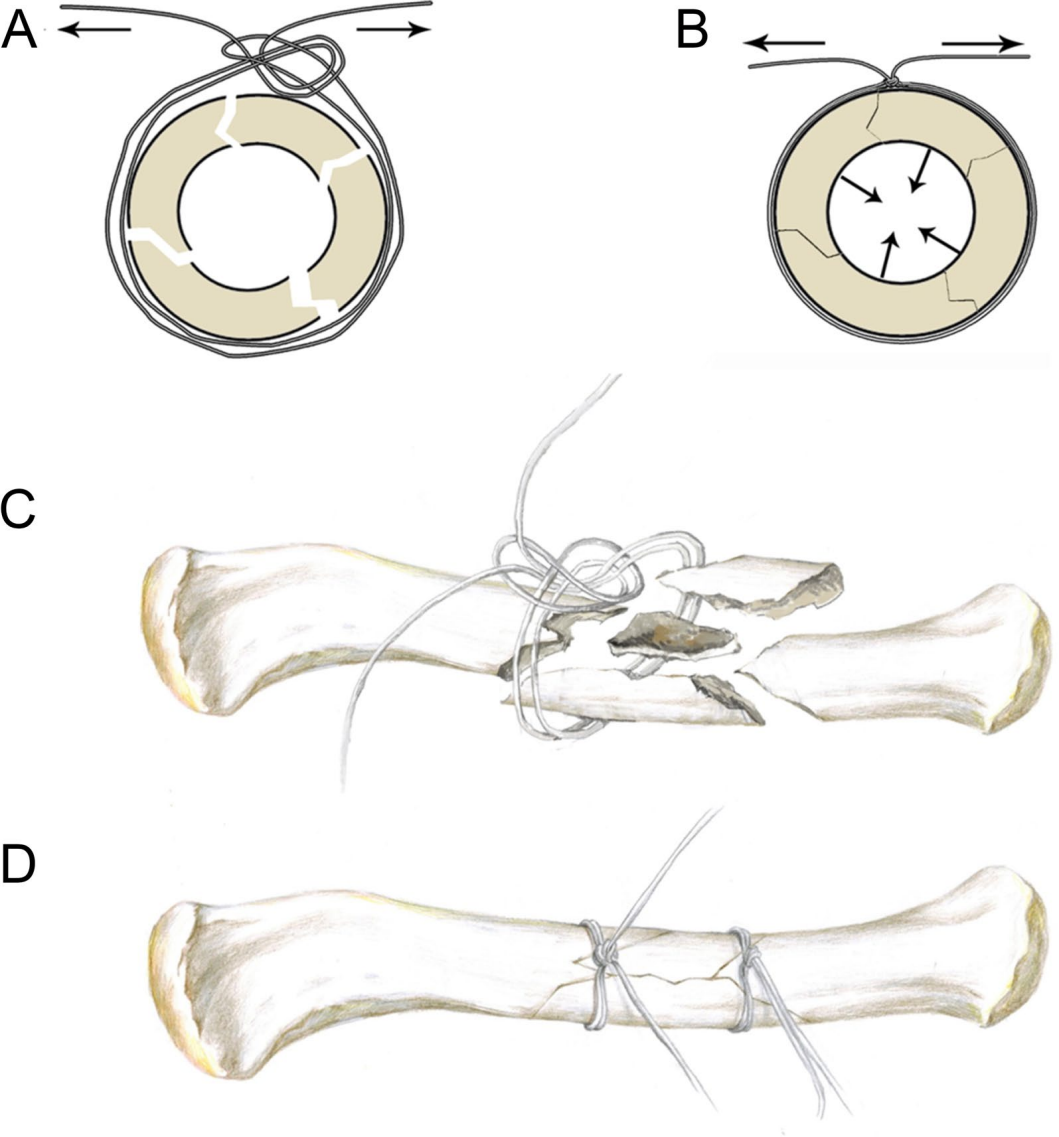
Schematic diagram of doubled-suture Nice knot in treating clavicle fracture. **A** and **C** The suture was doubled over itself to obtain two free limbs on one end and a loop on the other. The doubled-suture passed around the bone fragments and then the two free limbs went across the loop. **B** and **D** When the two free limbs of the suture were pulled apart, the bone fragment could be reduced and tied firmly, and the doubled-suture Nice knot could generate radial compression force within the bone fragments

The surgical procedures were as followed. After anesthesia, the patient was placed in supine position. The shoulder of the operative side was raised with a pad. A transverse incision along the clavicle was made to expose the fracture. After displacement of the bone fragments was reduced, some doubled-suture Nice knots (2 − 0 ETHIBOND EXCEL® Polyester Suture) were applied to fix the bone fragments. The knot was done as previously described [10]. The suture was doubled over itself to obtain two free limbs on one end and a loop on the other, and passed around the bone fragments by a needle. The needle should be attached closely to the bone to avoid neurovascular injury, as shown in Fig. 1A and C. When the two free limbs of the suture were pulled apart, bone fragments could be reduced and tied firmly, which was shown in Fig. 1B and D. The knot could lock itself and was not easy to be loosened again, which was the most unique advantage of doubled-suture Nice knot. After that, a proper length of locking plate was placed above the clavicle and several screws were applied to fix the clavicle fracture.

The shoulder of all patients was immobilized with an arm sling for 4 weeks. Limited shoulder mobilization was allowed at the initial 2 weeks post operation. In this period, the active flexion, extension and abduction of the shoulder should not be over 45 degrees. At 3–4 weeks post operation, 60 degrees of flexion, extension and abduction was allowed.

Medical and operation records were reviewed for all patients. The number of bone fragments was calculated from the 3D reconstruction image of computer tomography scan. The operation time, length of incision, fracture side, and blood loss during operation were recorded. At each outpatient visit, patients were assessed clinically and radiographically. Radiographic union was defined as complete cortical bridging between proximal and distal fragments, which was determined by the surgeons. Complication and failure of internal fixation were recorded. Disabilities of the arm, shoulder and hand (DASH) questionnaire was used for the assessment of the upper extremity function and was done by the patient at the last follow-up. University of California Los Angeles (UCLA) scores were applied to evaluate the shoulder functional outcome by the physician at the last follow-up.

### Statistical analysis

Statistical analysis was performed using SPSS 19.0 (SPSS Inc., Chicago, IL, USA). Data were presented as means ± standard deviation. Due to the retrospective observative nature of this study, all values have to be seen as explorative and descriptive.

## Results

### Preoperative assessment

A total of 56 patients who had unilateral comminuted midshaft clavicle fractures were included. The number of male patients was 38 (67.9 %) and of the female patients was 18 (32.1 %). The average age of patients was 47.89 ± 16.5 years. 25 fractures happened on the left side and 31 happened on the right side. The mean time between the surgery and the injury was 4.5 days after injury (range, 1–10 days). The clavicle fractures were classified according to the AO/OTA fracture and dislocation classification compendium-2018. The fracture characteristics were shown in Table 1.

**Table 1:** Demographic data of fracture characteristics

Demographic/classification	N = 56
*AO/OTA classification, n(%)*	
15.2 B	16 (28.6)
15.2 C	40 (71.4)
*Number of free bone fragments, n (%)*	
1	16 (28.6)
2	30 (53.6)
3	10 (17.9)

### Intraoperative situation

**Fig. 2:**
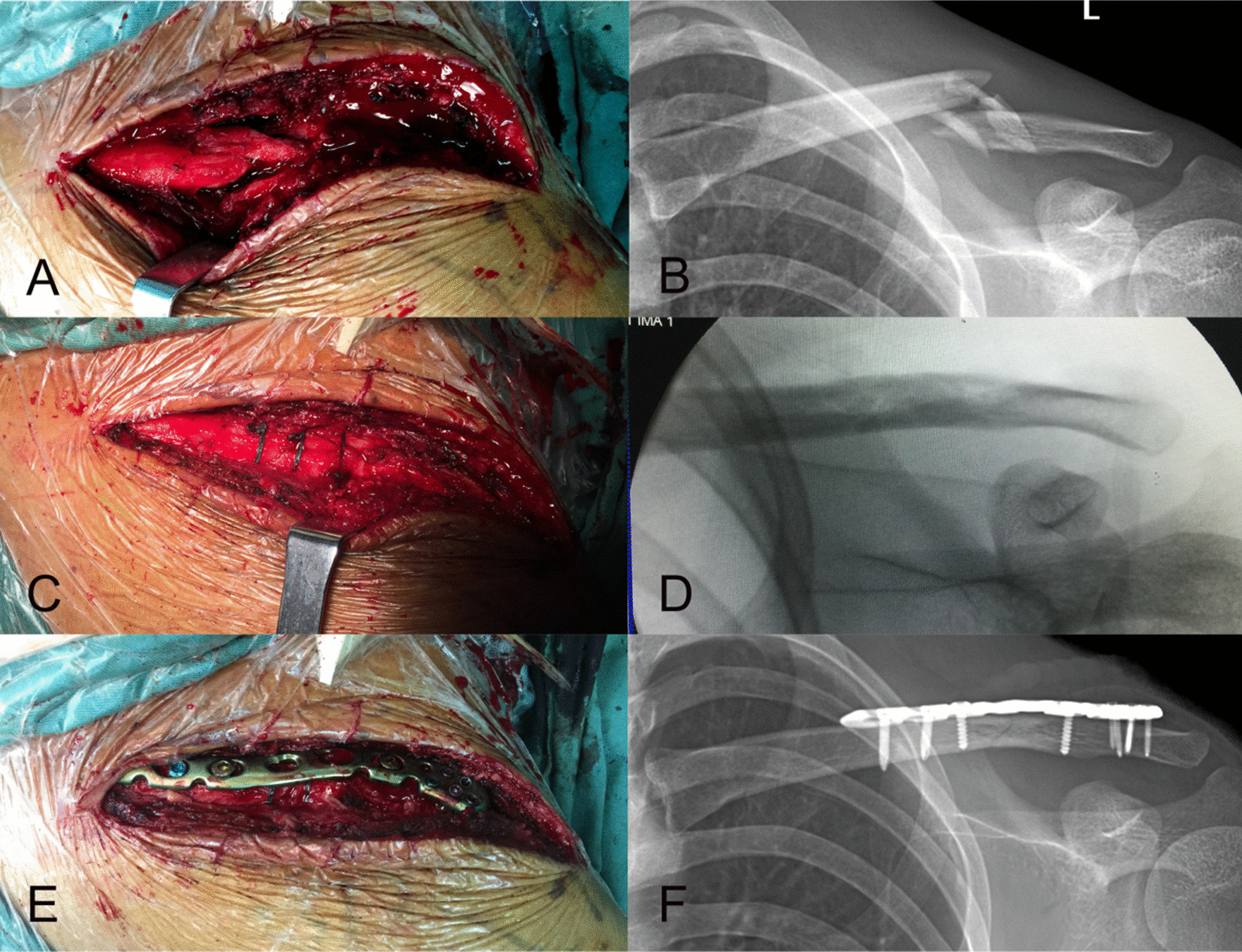
Intraoperative digital image and X-ray film of the clavicle fracture fixation. **A** Exposure of comminuted midshaft clavicle fracture. Many displaced bone fragments could be found within the fracture region, and the soft tissue attached to the bone fragments was maximally preserved. **B** Preoperative X-ray film of the clavicle. **C** Doubled-suture Nice knot fixed the fracture in anatomical reduction. **D** Intraoperative X-ray film revealed that the fracture achieved anatomical reduction after the clavicle was fixed with doubled-suture Nice knot. **E** A locking plate was subsequently applied to fix the fracture. **F** Postoperative X-ray film of the clavicle

All the patients were performed open reduction and doubled-suture Nice knot augmented plate fixation. After the fracture was exposed as shown in Fig. 2A, B, the bone fragments were encircled by the loop of doubled-suture Nice knot as shown in Fig. 2C, D. A periosteal detacher was applied to reduce the bone fragments, and then the doubled-suture Nice knot was tightened firmly. Most of the fracture could reach anatomical reduction after that. The periosteum of the bone fragments was also well protected in general. And then, locking plate-screws were applied to combine the fractures (Fig. 2E, F). The mean time of surgery was 85.6 ± 24.0 min. The average length of incision was 9.2 ± 1.9 cm. The number of doubled-suture Nice knots applied range from 1 to 5.

### Radiographic and functional outcomes

**Fig. 3:**
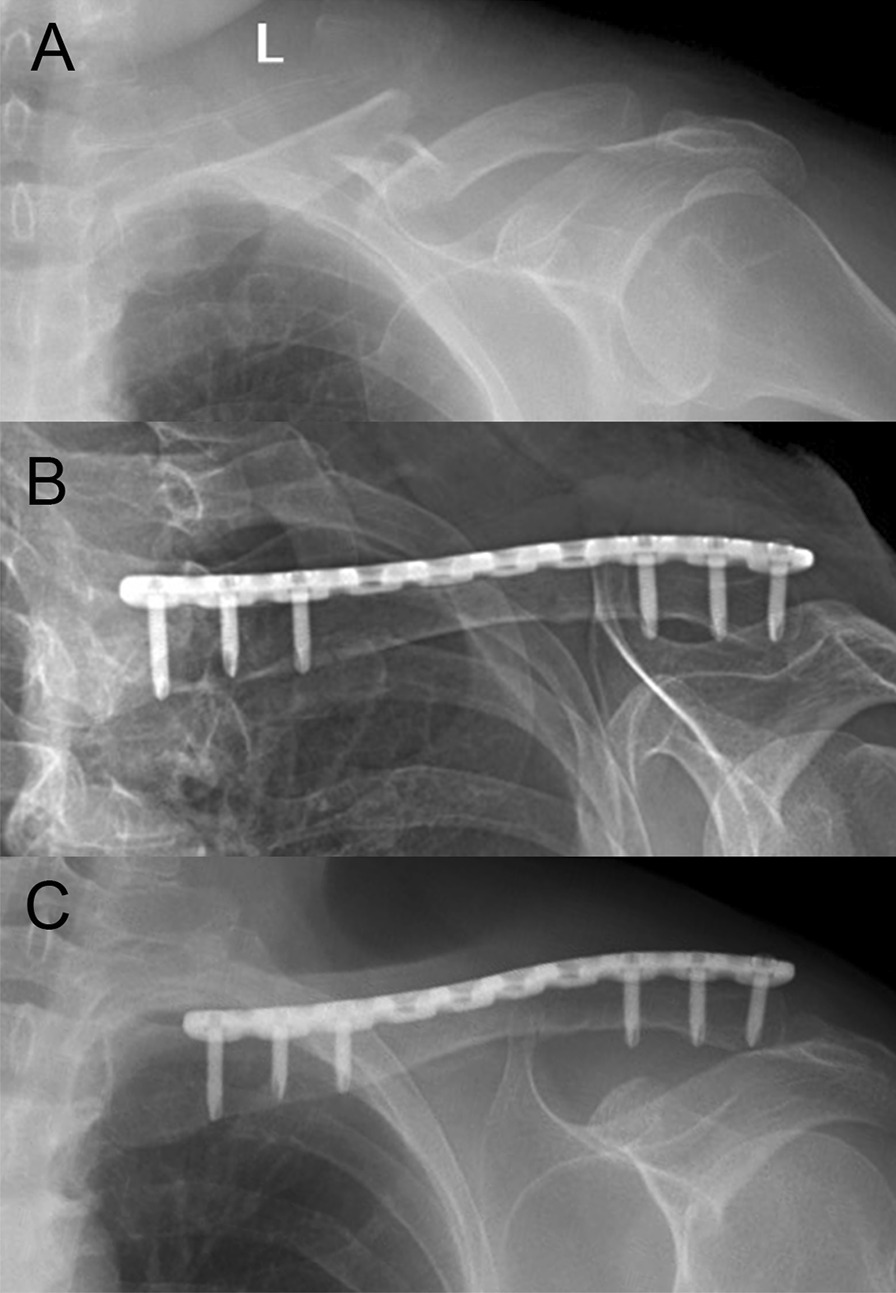
X-ray film of a 55-year-old female involved in a motorcycle accident. **A** Preoperative X-ray film indicated midshaft clavicle. **B** Immediate post-op X-ray film showed that the fracture was reduced. **C** X-ray film at three months after surgery shows that bone union was obtained

The mean time for follow-up was 25.6 months (range, 12–60 months). Immediate postoperative radiographs showed good fracture reduction, as shown in Fig. 3. For all patients, no screw loosening, failure of reduction, or metal plate breakage was observed. The radiographs at last follow-up revealed fracture union with abundant callus formation across the fracture sites and the disappearance of fracture line in all the patients. The mean time of fracture union was 19.8 weeks (range, 14–31 weeks). Most of the patients showed good functional outcome. The data of UCLA score and DASH score were shown in Table 2.

**Table 2:** Intraoperative parameters and follow-up data of the patients

Parameters	N = 56
Blood loss (ml, mean ± SD)	80.1 ± 42.1
Time of last follow-up (month, mean ± SD)	25.6 ± 7.6
Length of incision (cm, mean ± SD)	9.2 ± 1.9
Operation time (min, mean ± SD)	85.6 ± 24.0
Hospital stay (days, mean ± SD)	13.6 ± 6.1
Number of doubled-suture Nice knot applied, n (%)	
1	11 (19.6)
2	26 (46.4)
3	16 (28.6)
4	2 (3.6)
5	1 (1.8)
Implant failure, n (%)	0 (0 %)
DASH Score (mean ± SD)	0.86 ± 1.4
UCLA Score (mean ± SD)	34.1 ± 1.3

### Complications

All the patients reached bone union after the treatment. There was no neurovascular injury that happened intraoperatively. The operative wound healed finely, and no superficial or deep infection was observed. No implant failure was observed.

## Discussion

In this study, doubled-suture Nice knots were used for assisting the plate-screws fixation for comminuted midshaft clavicle fractures, and the efficacy and safety of the hybrid fixation were evaluated. We found that the application of doubled-suture Nice knots were very helpful for maintaining the reduction of bone fragments in the clavicle fracture. It could provide radically compressive force for the bone fragment, and restore the cortical bone defect to improve the rigidity of metal plate fixation for the midshaft clavicle fracture. Application of cerclage for long bone fracture could routinely raise concerns about bone nonunion issues. From the clinical outcomes of the subset of cases, we demonstrated that doubled-suture Nice knot did not affect the bone union of clavicle fractures. Thus, doubled-suture Nice knot augmented plate fixation could be a recommended approach for the treatment of comminuted mid shaft clavicle fracture.

The type and location of metal plate provided for clavicle fractures are factors that may affect the outcome of treatment. The complication rate is high and the biomechanical stability is low for reconstruction plate in treating midshaft clavicle fracture [12]. However, the rigidity of fracture fixation can be enhanced, and the risk of the screw loosening or malunion can be decreased when locking compression plate is used [13, 14]. Uzer et al. [15] revealed that the fixation strength of anterosuperior plating was greater than that of anterior plating under rotational forces, but as good as that of superior plating. Nevertheless, biomechanical analysis has proved that defect of cortical integrity of the fracture decreased the stiffness of plating [6]. Thus, the authors believe that both proper metal plate selection and fixation of free bone fragments are factors that can prevent fixation failure and loss of reduction.

The comminuted midshaft clavicle fractures often present with several small bone fragments. It is difficult to stabilize those bone fragments in the surgery. Some surgeons used dual plate fixation for acute midshaft clavicle fractures to improve the stiffness of fixation, resulting in excellent union rates and low complication rates [16, 17]. As we know, screws can only fix big bone fragments. However, in many comminuted fractures, the bone fragments are usually too small to allow screw fixation. Previously, we tried to use suture knots like square knots or surgical knots to aid the fixation of free bone fragments, but these knots could not provide sufficient compressive force to the bone fragments and were not easy to be tightened. The doubled-suture Nice knot combining a doubled suture with a sliding knot was self-stabilized and solid. It could be used to tie the bone fragments within the fracture site firmly, and finely solved the problem of free bone fragments fixation. The surgeons could use a mini periosteum detacher to clear the fracture site, and then surrounded the surface of the fragments with the doubled suture. When the suture was tightened, usually the bone fractures could be reduced automatically without excessive soft tissues detachment, which could ultimately restore the blood supply of butterfly fragments.

Application of cerclage in orthopedics has a long history. Although cerclage functioning as an exclusive implant is limited, it plays an important role in auxiliary fixation of a more rigid metal implant. Biomechanical studies have demonstrated that cerclages are especially suitable to fix radially displaced fracture fragments [18]. Most bone fragments of our patients displaced radially in comminuted clavicle fractures. After the fixation of doubled-suture Nice knots, most fractures could reach anatomical reduction. The knot could provide sufficient tension for the bone fragments and maintain the stability of the fracture fixation. No fixation failure or nonunion was observed, which indicated that the doubled-suture Nice knot was an effective auxiliary fixation to restore the cortical bone contact of the fracture. Blood supply to the bone is essential for bone healing. The effects of cerclage wire to the bone blood supply are still controversial. Although concerns for a strangled blood supply due to the application of cerclage are still present, several studies have also reported that cerclage wire does not restrict cortical vascularity as the area of contact is rather narrow [18, 19]. Moreover, during the healing of bone fractures in our research, the endosteal circulation also provides a majority of blood to the fracture region. No fracture in our research was treated with IM fixation, thus the endosteal circulation was preserved. As the clavicle fractures healed successfully, the authors believe that plate-screws fixation combined with the doubled-suture Nice knot may not affect the healing of bone fractures.

Application of doubled-suture Nice knot in the surgery requires that the fracture line should not be too vertical to the radial axis of the clavicle. Usually, clavicle fractures with a fracture line with 45° or above relative to the radial axis of the clavicle will be increasingly difficult or impossible to be treated with this method. However, most of clavicle fractures will be in 45° or below and therefore well suited for this treatment. Cerclage wiring has been reported to be used for assisting the intramedullary nailing for fractures and can reduce the risk of malunion [20]. Intramedullary nailing for fractures of middle third clavicle has become increasingly popular. Open nailing was reported to have similar results in terms of time to union and functional outcome when compared with close nailing [21]. We may have to do open nailing, when close nailing for middle third clavicle fracture is failed in the surgery. Doubled-suture Nice knot can be helpful to maintain the anatomic reduction of the clavicle fracture prior to nailing. In some non-comminuted midshaft clavicle fractures, especially when the fracture lines are very long in radial axis of the clavicle, doubled-suture Nice knot augmented plate fixation can also significantly improve the rigidity of fracture fixation in our experience. However, we tend to use lag screws, when the fracture lines are short in the radial axis of the clavicle.

This study had several limitations. No control group was included in this study, we majorly summarized the clinical outcomes of doubled-suture Nice knot aided plate-screw fixation for the treatment of comminuted midshaft clavicle fracture. Therefore, we should not attribute the good clinical outcome solely to the application of the doubled-suture Nice knot. The property of different types of knots in the fixation of comminuted clavicle fracture needed further evaluation via the biomechanical analysis. Moreover, no intraoperative adverse events or postoperative complications related to the fixation method were observed, indicating that the fixation method was safe. Nevertheless, the safety of doubled-suture Nice knot still needed further research due to the small sample size of our research.

## Conclusions

Doubled-suture Nice knot aided plate-screw fixation showed good functional outcomes, and no implant failure or bone nonunion was observed in our study. Therefore, combination of doubled-suture Nice knot and plate-screws fixation was a reliable method in treating comminuted midshaft clavicle fractures.

## Data Availability

The datasets used and/or analyzed during the current study available from the corresponding author on reasonable request.

## Abbreviations

IM: Intramedullary
DASH score: The arm, shoulder and hand questionnaire
UCLA score: The University of California Los Angeles scores

## References

[1] Altamimi SA, McKee MD (2008). Nonoperative treatment compared with plate fixation of displaced midshaft clavicular fractures. Surgical technique. J Bone Jt Surg American volume.

[2] Hulsmans MH, van Heijl M, Houwert RM, Burger BJ, Verleisdonk EJM, Veeger DJ (2018). Surgical fixation of midshaft clavicle fractures: a systematic review of biomechanical studies. Injury.

[3] Boonard M, Sumanont S, Arirachakaran A, Sikarinkul E, Ratanapongpean P, Kanchanatawan W (2018). Fixation method for treatment of unstable distal clavicle fracture: systematic review and network meta-analysis. Eur J Orthop Surg Traumatol.

[4] Erdle B, Izadpanah K, Jaeger M, Jensen P, Konstantinidis L, Zwingmann J (2017). Comparative analysis of locking plate versus hook plate osteosynthesis of Neer type IIB lateral clavicle fractures. Arch Orthopaed Trauma Surg.

[5] Fritz EM, van der Meijden OA, Hussain ZB, Pogorzelski J, Millett PJ (2017). Intramedullary Fixation of Midshaft Clavicle Fractures. J Orthopaed Trauma.

[6] Drosdowech DS, Manwell SE, Ferreira LM, Goel DP, Faber KJ, Johnson JA (2011). Biomechanical analysis of fixation of middle third fractures of the clavicle. J Orthopaed Trauma.

[7] Wilson DJ, Scully WF, Min KS, Harmon TA, Eichinger JK, Arrington ED (2016). Biomechanical analysis of intramedullary vs. superior plate fixation of transverse midshaft clavicle fractures. J Shoulder Elbow Surg.

[8] Meeuwis MA, Pull Ter Gunne AF, Verhofstad MH, van der Heijden FH (2017). Construct failure after open reduction and plate fixation of displaced midshaft clavicular fractures. Injury.

[9] Batash R, Debi R, Grinberg D, Shema M, Elbaz A, Benedict Y (2019). Mechanical failure of plate breakage after open reduction and plate fixation of displaced midshaft clavicle fracture—a possible new risk factor: a case report. J Med Case Rep.

[10] Boileau P, Alami G, Rumian A, Schwartz DG, Trojani C, Seidl AJ (2017). The doubled-suture Nice knot. Orthopedics.

[11] Hill SW, Chapman CR, Adeeb S, Duke K, Beaupre L, Bouliane MJ (2016). Biomechanical evaluation of the Nice knot. Int J Shoulder Surg.

[12] Chiu YC, Huang KC, Shih CM, Lee KT, Chen KH, Hsu CE (2019). Comparison of implant failure rates of different plates for midshaft clavicular fractures based on fracture classifications. J Orthop Surg Res.

[13] Eden L, Doht S, Frey SP, Ziegler D, Stoyhe J, Fehske K (2012). Biomechanical comparison of the Locking Compression superior anterior clavicle plate with seven and ten hole reconstruction plates in midshaft clavicle fracture stabilisation. Int Orthop.

[14] Pai HT, Lee YS, Cheng CY (2009). Surgical treatment of midclavicular fractures in the elderly: a comparison of locking and nonlocking plates. Orthopedics.

[15] Uzer G, Yildiz F, Batar S, Bozdag E, Kuduz H, Bilsel K (2017). Biomechanical comparison of three different plate configurations for comminuted clavicle midshaft fracture fixation. J Shoulder Elbow Surg.

[16] Chen X, Shannon SF, Torchia M, Schoch B (2017). Radiographic outcomes of single versus dual plate fixation of acute mid-shaft clavicle fractures. Arch Orthopaed Trauma Surg.

[17] Yanev P, Zderic I, Pukalski Y, Enchev D, Rashkov M, Varga P, et al. Two reconstruction plates provide superior stability of displaced midshaft clavicle fractures in comparison to single plating—biomechanical study. Clin Biomech (Bristol, Avon). 2020:105199. 10.1016/j.clinbiomech.2020.105199 33129563

[18] Perren SM, Fernandez Dell’Oca A, Lenz M, Windolf M (2011). Cerclage, evolution and potential of a Cinderella technology. An overview with reference to periprosthetic fractures. Acta Chirurgiae Orthopaedicae et Traumatologiae Cechoslovaca.

[19] Angelini A, Battiato C (2015). Past and present of the use of cerclage wires in orthopedics. Eur J Orthopaed Surg Traumatol Orthopedie Traumatologie.

[20] Kennedy MT, Mitra A, Hierlihy TG, Harty JA, Reidy D, Dolan M (2011). Subtrochanteric hip fractures treated with cerclage cables and long cephalomedullary nails: a review of 17 consecutive cases over 2 years. Injury.

[21] Saraf H, Kasture S (2016). Closed vs open nailing for displaced middle third fracture of clavicle. Does it matter?. J Clin Orthop Trauma.

